# Amino Acid and Peptide Immobilization on Oxidized Nanocellulose: Spectroscopic Characterization 

**DOI:** 10.3390/nano2020187

**Published:** 2012-06-12

**Authors:** Saïd Barazzouk, Claude Daneault

**Affiliations:** Centre de Recherche sur les Matériaux Lignocellulosiques, Université du Québec à Trois-Rivières, Trois-Rivières, QC, G9A 5H7, Canada; Email: claude.daneault@uqtr.ca

**Keywords:** oxidized nanocellulose, amino acids, peptides, amide bond, spectroscopic characterization

## Abstract

In this work, oxidized nanocellulose (ONC) was synthesized and chemically coupled with amino acids and peptides using a two step coupling method at room temperature. First, ONC was activated by *N*-ethyl-*N’*-(3-dimethylaminopropyl) carbodiimide hydrochloride, forming a stable active ester in the presence of *N*-hydroxysuccinimide. Second, the active ester was reacted with the amino group of the amino acid or peptide, forming an amide bond between ONC and the grafted molecule. Using this method, the intermolecular interaction of amino acids and peptides was avoided and uniform coupling of these molecules on ONC was achieved. The coupling reaction was very fast in mild conditions and without alteration of the polysaccharide. The coupling products (ONC-amino acids and ONC-peptides) were characterized by transmission electron microscopy and by the absorption, emission, Fourier transform infrared spectroscopy (FTIR) and X-ray photoelectron spectroscopy (XPS) spectroscopic techniques.

## 1. Introduction

Cellulose is the most abundant natural polymer found in nature occurring in wood, cotton, hemp and other plant-based materials. It is also synthesized by algae, tunicates, and some bacteria [1,2,3]. Recently, considerable interest has been focused on finding new applications for this biopolymer. One of these applications has been the preparation and development of novel biocomposites based on nanocellulose. 

Natural celluloses are complex hierarchical structures, biosynthesized and self-assembled into microfibrils having diameters ranging from 2 nm to 20 nm and lengths ranging from 100 nm to several micrometers depending on their biological origin [4,5]. These microfibrils are hooked very tightly to one another by multiple hydrogen bonds such that their extraction has proven extremely difficult. However, the microfibrils can be individualized into nanocellulose by different methods such as mechanical treatment, acid hydrolysis, and catalyzed oxidation [6,7,8,9,10,11,12,13,14]. Unfortunately mechanical treatment requires a large amount of energy, and the resulting products consist mainly of bundles of microfibrils [6,15], while acid hydrolysis depolymerizes the cellulose chains and yields a dramatic decrease in the microfibrils length and width which is not advantageous since it damages the structure of microfibrils [4,14,16]. Thus, it has not been possible to obtain individualized cellulose microfibrils using these two techniques without altering their structural properties. 

To bypass these problems, the promising route toward the preparation of suspensions of nanocellulose depends on the catalyzed oxidation of cellulose. This technique is based on the selective oxidation of primary alcohol groups of polysaccharides catalyzed by 2,2,6,6-tetramethyl-1-piperidine oxoammonium radical (TEMPO) in presence of NaOBr (generated in situ by NaOCl and NaBr) which helps regenerate the catalyst TEMPO during the reaction. Using this oxidative reaction, it was found that cellulose could be completely converted into water-soluble polyglucuronic acid [17,18]. In the case of native cellulose fibers, the oxidation proceeded throughout the fibers but occurred only at the surface of the microfibrils, which therefore became negatively charged [19]. The chemical modification is a way to introduce specific functionalities for developing new nanomaterials (biopolymers). In this context, the oxidized cellulose containing carboxylic groups can serve as templates to bind different molecules of interest (fluoprobes, peptides, antibodies). Thus, the preparation of novel biocomposites based on oxidized nanocellulose will open new areas for applications in food industry, cosmetics, medicine, packaging, and other areas. 

In the present work, effort has been made to probe the coupling of two fluorescent amino acids L-Tryptophan (Trp) and L-Phenylanaline (Phe), and two Trp-based peptides Endomorphin-1 (EMP) and W-Nle-R-F-NH_2_ (WRF) (Table 1) onto oxidized nanocellulose (ONC). Hereafter, the amino acid and Trp-based peptide grafted onto ONC will be referred to as ONC-Trp, ONC-Phe, ONC-EMP and ONC-WRF. The choice of fluorescent amino acids and peptides in this study is to further characterize their coupling with ONC using fluorescence spectroscopy in addition to other techniques. The advantage in using ONC rather than other cellulose products is that the coupling reaction between ONC and the amino acid or peptide is very fast in mild conditions and without alteration of the polysaccharide [20]. This coupling is a nucleophilic reaction between the amine groups (–NH_2_) of the amino acid or peptide and carboxyl group (–COOH) of the ONC, and is commonly catalyzed by the coupling agent hydrochloride 1-ethyl-3-(3-dimethylaminopropyl) carbodiimide (EDAC) in the presence of an activation agent such as *N*-hydroxysuccinimide (NHS) or 1-hydroxybenzotriazole (HOBt) [21]. Further details regarding the regioselective amidation between carboxylic acid groups and amino groups can be found elsewhere [22,23,24,25,26]. The extent of coupling was investigated by TEM, and by absorption, fluorescence, FTIR and XPS spectroscopic techniques.

**Table 1:** Sequence and structure of amino acids and Trp-based peptides.

Chemicals	Sequence	Structure
**L-Tryptophan; (Trp)**	Trp	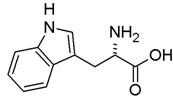
**L-Phenylanaline; (Phe)**	Phe	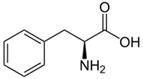
**Endomorphin-1; (EMP) **	Tyr-Pro-Trp-Phe-NH_2_	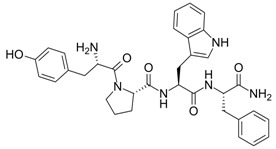
**W-Nle-R-F-NH_2_; (WRF)**	Trp-Nle-Arg-Phe-NH_2_	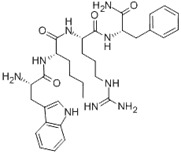

## 2. Results and Discussion

### 2.1. Absorption Spectra

**Figure 1:**
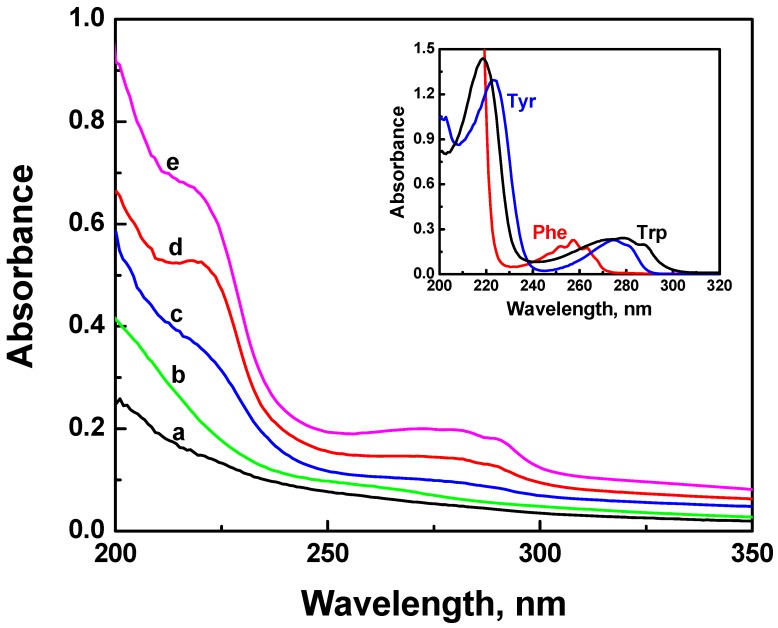
Absorption spectra of aqueous suspension of ONC: (**a**) alone, and coupled with: (**b**) Phe, (**c**) EMP, (**d**) Trp and (**e**) WRF. The insert shows the absorption spectrum of Trp (35.1 μM), Phe (1.2 mM) and Tyr (1.2 mM) in deionized water.

The amino acids tryptophan (Trp), tyrosine (Tyr), and phenylalanine (Phe) are the molecules responsible for the absorption and fluorescence of peptides in the UV region. Figure 1 presents the absorption spectra of aqueous suspensions of ONC before and after its coupling with different amino acids and Trp-based peptides (Trp-Ps) at pH 7–8. The absorption spectrum of ONC alone (spectrum a) shows no characteristic absorption bands. Upon coupling of ONC with amino acids or Trp-Ps, new absorption bands (219 nm and a large band centered at ~ 280 nm) are present in the absorption spectra of all samples except that of ONC-Phe. These absorption bands are clearly visible in the absorption spectra of ONC-EMP, ONC-Trp and ONC-WRF (spectra c–e, respectively) and are characteristic of the principal absorption peaks of Trp in deionized water (see insert of Figure 1). The absorption spectrum of ONC-Phe (Figure 1, spectrum b) shows broad absorption bands in 220 and 260 nm region corresponding to the absorption of Phe (~257 nm, inset of Figure 1). The absorption spectra presented in Figure 1, suggest that there is a good coupling of ONC with WRF and Trp but not with other samples. 

The extent of the coupling of ONC with Trp and Phe was evaluated by determining: (i) the degree of oxidation of ONC before (DO) and after (DO_1_) coupling with the amino acid; and (ii) the degree of coupling (DC) using an electric conductivity titration method [26]. The coupling yield (CY) was then calculated using the following equation:

CY (%) = (DC/ DO) × 100 (1)

where DC = DO – DO_1_. 

The degree of oxidation of ONC before the grafting is DO = 0.22. The values of DO_1_ and CY (%) for ONC-Trp and ONC-Phe are summarized in Table 2. As seen from this Table, ONC-Trp shows a higher coupling yield. 

**Table 2:** DO_1_ and CY (%) for ONC-Trp and ONC-Phe obtained by conductometric titration.

	ONC-Trp	ONC-Phe
**DO_1_**	0.17	0.19
**CY (%)**	23	14

The efficiency of the coupling of ONC with EMP and WRF can not be determined using the electric conductivity titration method because of the structure of the peptide that contains different amino groups which can greatly influence the titration results. However, we can use the absorption spectra of ONC-EMP and ONC-WRF in Figure 1 to have an idea on the efficiency of the coupling reaction between peptides and ONC. Thus, the extent of coupling of ONC with WRF and EMP was deduced by simple calculation of the number of Trp-Ps, *i.e.*, the concentration c, attached to the ONC using the Beer-Lambert law: 

Amax = εmax c (Trp − Ps) × l (2)

where A_max_ is the maximum absorption peptide, ε_max_ its extinction coefficient at the maximum absorption, and l is the path length of the cell (= 1 cm). The average concentration of the coupled peptide calculated using the Beer-Lambert law was: 7.5 and 26.6 μM for ONC-EMP and ONC-WRF, respectively. Based on the initial concentration of the added peptides, *i.e.*, 117 and 115 μM for EMP and WRF, respectively, one can deduce the percentage of the grafted peptides onto ONC to be: 6% and 23% for ONC-EMP and ONC-WRF, respectively. Thus, one can notice the good coupling of ONC with WRF compared to that with EMP. It should be mentioned that the absorption spectra of ONC and ONC-Trp-Ps are influenced by the widths of the ONC suspension as described by Carr and Hermans [27]. This influence has been taken into account when we have calculated the percentages of the grafted peptides onto ONC based on their absorption spectra (Figure 1). This has been achieved by subtracting the absorption spectrum of ONC alone from the absorption spectra of grafted ONC.

We have also studied the effect of the pH on the coupling reaction of ONC with amino acids and Trp-Ps by carrying out the coupling reaction at different pH. At pH lower than 7, no absorption bands were observed in the absorption spectra of ONC-amino acids or ONC-Trp-Ps indicating that none of the amino acids or Trp-Ps has been attached to ONC. These results were due to the instability of the coupling agent EDAC at lower pH leading to the instability of the esterified ONC during the first step of the coupling reaction between ONC and the amino acids or Trp-Ps [28,29]. At pH higher than 10, the same result (no coupling) was obtained. In fact, Danishefsky and Siskovic [28] have reported that at higher pH the coupling reaction becomes slower. This demonstrates that the grafting of ONC with amino acids and Trp-Ps or other peptides is favorable at pH between 7 and 10.

### 2.2. TEM Images

**Figure 2:**
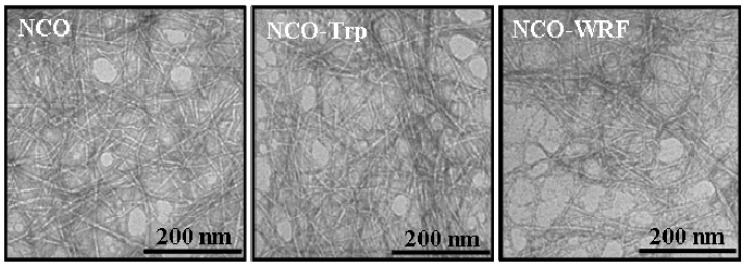
Transmission electron micrographs of ONC, ONC-Trp and ONC-WRF.

Figure 2 shows TEM images of ONC alone and grafted with Trp and WRF from transparent suspensions. TEM images of ONC-Phe and ONC-EMP were very similar to those of ONC-Trp and ONC-WRF, and thus there are not shown in Figure 2. Individualized nanofibrils with shapes and sizes similar to those reported by Saito *et al.*, [11,30] were obtained. 

Using an image processing software, the average width of the nanofibrils of all samples was between 4 nm and 6 nm. The small width makes the ONC suspension transparent and significantly viscous even at very low solid fraction. However, no solitary nanofibrils could be identified in order to determine their lengths. One can also notice from all micrographs the presence of kinks on the microfibrils which are probably due to the mechanical treatment used during the preparation of ONC. After the grafting of ONC with amino acids or Trp-based peptides, Figure 2 shows that the morphology of ONC fibers did not change. 

### 2.3. Fluorescence Properties of ONC and Grafted ONC

The emission of cellulose from different sources (wood pulp, cotton, bacteria and algae) has been studied in detail by Olmstead and Gray [31]. Upon their excitation with 320 nm light, all cellulose samples displayed a characteristic fluorescence emission, with a maximum of 420 nm and a shoulder at 390 nm. They also concluded that the presence of carbonyl groups decreases the fluorescence emission of cellulose, whereas the reduction of these groups to alcohols increases fluorescence intensity. 

**Figure 3:**
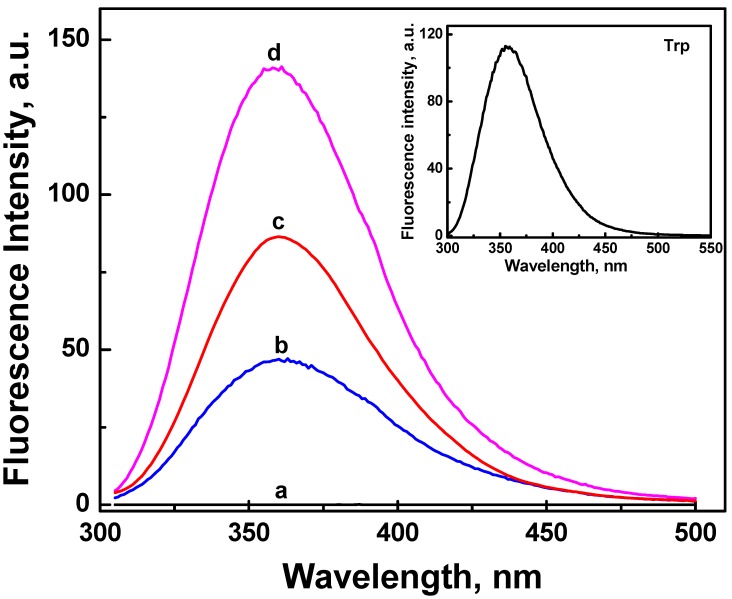
Fluorescence emission spectra of aqueous suspension of ONC: (**a**) alone, and coupled with: (**b**) Trp-EMP, (**c**) Trp and (**d**) Trp-WRF. The insert shows the emission spectrum of an aqueous solution of Trp. The excitation wavelength was 295 nm.

In Figure 3, ONC alone (spectrum a) shows a very weak or almost no fluorescence intensity in the UV region. This lack of fluorescence emission is most probably due to the quenching effect caused by the presence of carbonyl and carboxyl groups on ONC. Further, the oxidizing agents (TEMPO and NaOCl) used during the oxidation process of cellulose are potential quenchers of cellulose emission. In fact, Toner and Plitt [32] have reported that oxidizing agents induced a decrease in fluorescence intensity of cotton-cellulose by creating carbonyl groups.

Tryptophan fluorescence is very sensitive to the local environment. In an environment with a low polarity, tryptophan emits at a maximum of 320 nm. The peak position shifts to 355 nm in the presence of a more polar environment. Also, upon binding of a ligand to a protein, Trp fluorescence characteristics (intensity, polarization, and lifetime) can be altered, and so one can follow this binding with Trp fluorescence. In proteins, tryptophan fluorescence dominates. Zero or weak fluorescence of tyrosine and phenylalanine is the result of energy transfer from these molecules to tryptophan and/or neighbouring amino acids [33]. 

The absorption spectra of the aromatic amino acids Trp, Tyr and Phe, overlap at many wavelengths. Since we want to study the emission from the Trp residue alone, we have chosen 295 nm as excitation wavelength for all samples in order to selectively excite Trp residue, thus avoiding the excitation of Tyr and Phe. As a result, the energy transfer from tyrosine to tryptophan does not take place, and the emission observed emanates solely from the tryptophan residue. 

The fluorescence spectra of aqueous suspensions of grafted ONC are also displayed in Figure 3. The fluorescence emission spectra of ONC-Trp, ONC-EMF, and ONC-WRF (spectra b–d) show a maximum emission at ~360 nm. This emission band originates mainly from the excitation of Trp residue (see the inset of Figure 3). In deonized water Trp has a maximum emission at 357 nm. We can also notice a 3 nm red-shift of the grafted ONC emission maxima (360 nm) compared to that of Trp alone (357 nm). This shift strongly suggests the grafting of Trp, EMP and WRF on ONC. It should be mentioned that in absence of the coupling agents (EDAC and NHS), all grafted ONC samples exhibit a very weak fluorescence similar to that of ONC alone (spectrum a in Figure 3), indicating that Trp, EMP and WRF have not grafted onto ONC in absence of coupling agents as it should be. 

**Figure 4:**
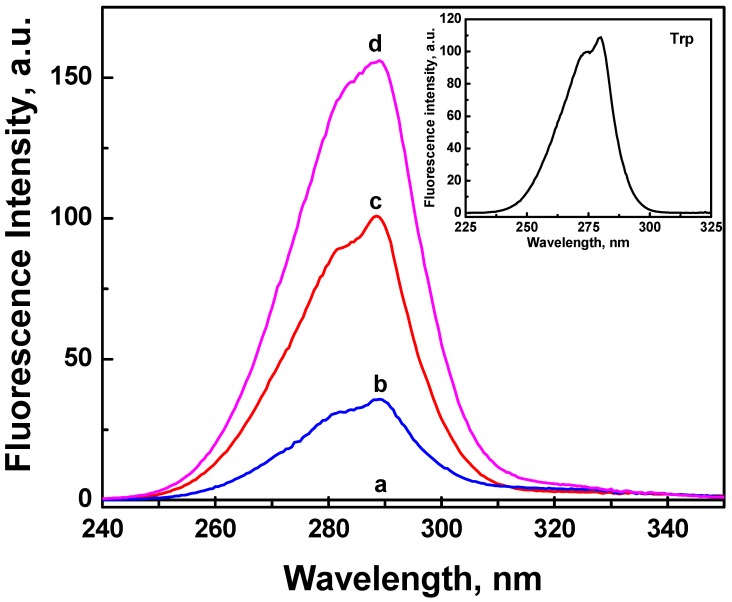
Fluorescence excitation spectra of aqueous suspension of ONC: (**a**) alone, and coupled with: (**b**) Trp-EMP, (**c**) Trp and (**d**) Trp-WRF. The insert shows the excitation spectrum of an aqueous solution of Trp. The emission was monitored at 375 nm.

We have also recorded the fluorescence excitation spectra for all samples (Figure 4). The excitation spectrum of ONC alone (spectrum a, Figure 4) shows a very weak signal, but upon its grafting with Trp or Trp-based peptides, two bands with maximum at 282 and 289 nm were observed. Comparing theses excitation spectra with the excitation spectrum of Trp alone shown in the insert of Figure 4, one can deduce that the fluorescence emission spectra for the grafted ONC shown in Figure 3 originate from the excitation of the Trp residue. 

### 2.4. FTIR Experiments

**Figure 5:**
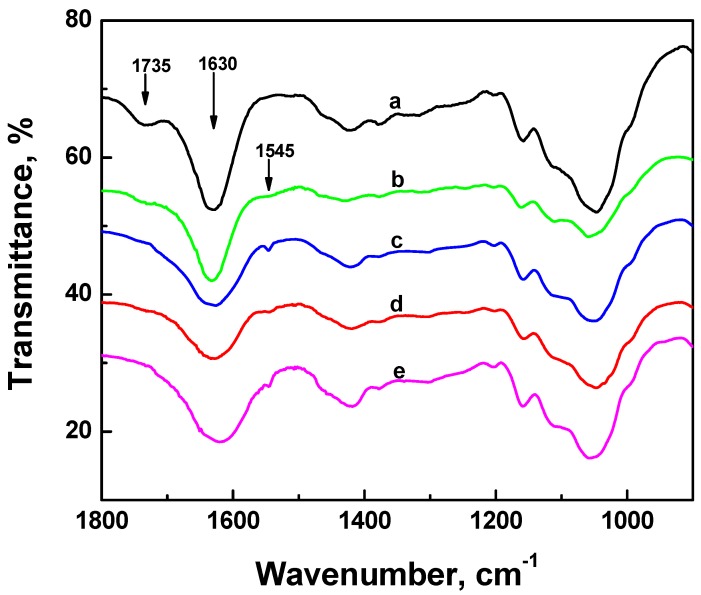
FTIR spectra of ONC: (**a**) alone, and coupled with: (**b**) Phe, (**c**) EMP, (**d**) Trp and (**e**) WRF.

**Scheme 1:**
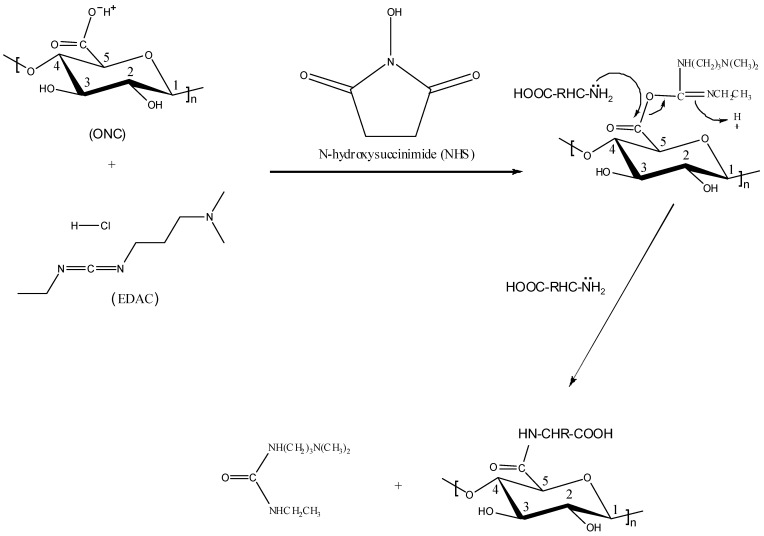
Illustration of the coupling mechanism between oxidized nanocellulose (ONC) and an amino acid (H_2_N–CHR–COOH, where R is an organic substituent).

Figure 5 shows the FTIR spectra of the ONC before and after its grafting with different amino acids and peptides. Spectrum a in Figure 5 shows one peak at 1735 cm^−1^ corresponding to the C=O band of the carboxylic acid of ONC which confirms the oxidation of native cellulose [26]. The second band at 1630 cm^−1^ represents the OH bending of the adsorbed water [34]. After grafting of ONC with amino acids and peptides, one can observe the disappearance of its acid band at 1735 cm^−1^ (Figure 5, spectra b–e). This change can be explained by the formation of an amide bond between the acid group of ONC and the amino group of the amino acid or peptide as a result of the coupling reaction (Scheme 1). The FTIR absorption band of the C–N bond of the amide appears as a small shoulder at ~1545 cm^−1^. It should be noted that the FTIR absorption band of C=O of the amide (HN–C=O) at 1630–1680 cm^−1^ region was not observed in the present experiment because it was masked by the strong absorption band of the adsorbed water in this region. The other FTIR absorption bands of all the samples were almost similar, and are comparable to those obtained by other researchers [26,35,36].

### 2.5. XPS Results

**Figure 6:**
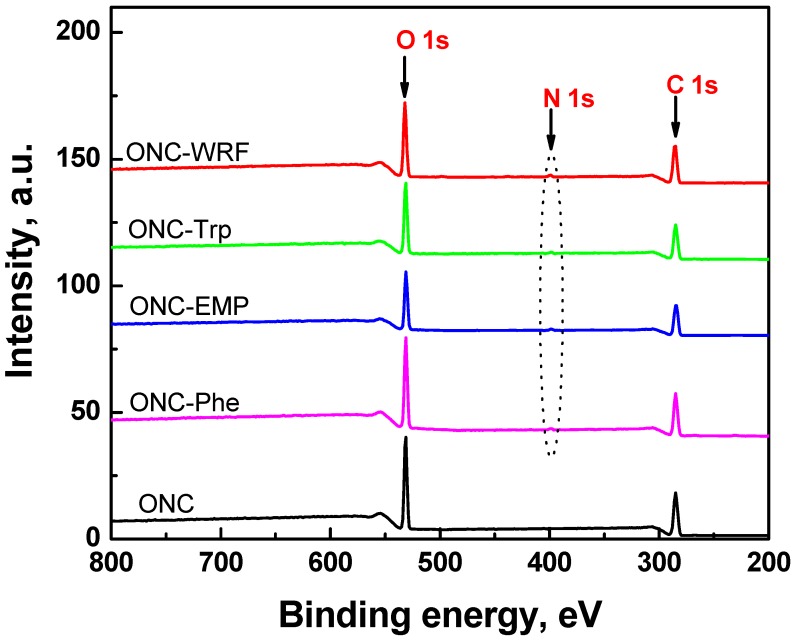
XPS survey spectra of ONC, ONC-Phe, ONC-EMP, ONC-Trp and ONC-WRF.

To further elucidate the extent of the grafting of ONC with amino acids and peptides, XPS study has been carried out. Figure 6 shows the XPS survey spectra for ONC and coupled ONC samples. Table 3 lists the experimental atomic composition as determined from the XPS spectra analysis, and the calculated oxygen to carbon (O/C) ratio for all samples. All XPS spectra reveal that C and O are the predominant species and they occur at 285 eV and ~533 eV, respectively. The presence of nitrogen on the surface was detected from its characteristic emission peak at ~400 eV and is due to the grafting of different amino acids or peptides on ONC (Figure 6). We have also recorded the XPS spectra of grafted ONC samples prepared in the absence of coupling agents EDAC and NHS (results not shown). In this case, the spectra did not show any nitrogen signal confirming the absence of amino acids and Trp-Ps from the ONC surface. 

The analysis of the data presented in Table 3 shows an increase in the percentage of carbon atoms and a considerable decrease in the percentage of oxygen atoms for grafted ONC samples compared to that of ONC alone. The most important change was observed for the nitrogen content which increased from 0% for ONC alone to up to 1.7% upon grafting of WRF on ONC. Table 3 also shows O/C ratios of different samples derived from their low-resolution XPS spectra and represent the average of three experimental data for each sample. In the present study, we have obtained an O/C ratio of 0.58 for ONC. This value is in agreement with the reported values in the literature. For example, Freire *et al.*, [37] have reported O/C values which vary between 0.75 and 0.12 for untreated and treated cellulose fibers, respectively. Also, Matuana *et al.*, [38] have found O/C values varying between 0.52 and 0.59 for untreated wood fibers. The O/C value of ONC (0.58) is, however, lower than that (0.83) for pure cellulose reported in the literature [38,39]. This difference in the O/C value between ONC and pure cellulose is due to the nanomaterial character of ONC in addition to the effects of different chemical and mechanical treatments used to obtain ONC from the native cellulose fibers (never-dried bleached kraft pulp). The O/C ratio decreases further from 0.58 to 0.46 upon grafting of ONC as a result of the partial removal of oxygen during the formation of the amide bond between the carboxylic group of ONC and the amino group of the amino acid or peptide (Scheme 1). 

**Table 3:** Experimental atomic composition and O/C ratio obtained by XPS analysis for ONC, ONC-Trp, ONC-Phe, ONC-EMP, and ONC-WRF. Errors in determination ± 1%.

Sample	Atomic content (%)			O/C
	C	O	N	
**ONC**	63.8	36.2	0	0.57
**ONC-Trp**	64.4	34.2	1.4	0.53
**ONC-Phe**	64.0	33.9	1.1	0.53
**ONC-EMP**	67.2	31.3	1.5	0.47
**ONC-WRF**	67.1	31.2	1.7	0.46

**Figure 7:**
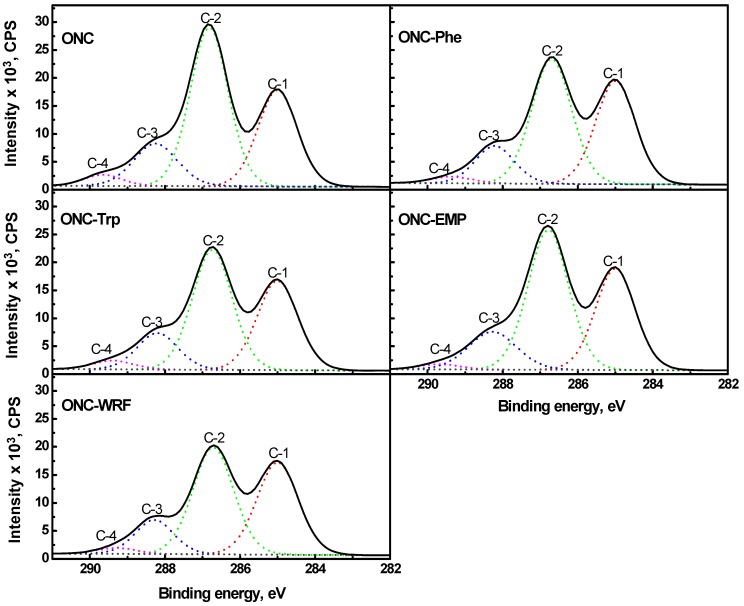
C 1s narrow scan XPS spectra for ONC, ONC-Phe, ONC-EMP, ONC-Trp and ONC-WRF.

In order to obtain more XPS information, high-resolution scans of the XPS spectra of C1s, O1s and N1s levels with their respective deconvolutions were also obtained. Figure 7 shows that the deconvolution of the C1s levels gives four peaks (C-1, C-2, C-3 and C-4) for all the samples which are in accordance with most data of the literature [40,41,42,43,44,45,46]. The C-1 (285 eV) corresponds to a carbon atom bound only to other carbon atoms and/or hydrogen atoms. The C-2 (286.7–286.8 eV) corresponds to a carbon bound to a single noncarbonyl oxygen atom, which has been shown to be mainly derived from cellulose [47]. The C-3 peak (288.2–282.3 eV) represents a carbon atom bound to one carbonyl oxygen or to two noncabonyl oxygen atoms. The C-4 (289.3–289.5 eV) represents a carbon atom linked to one carbonyl oxygen and one noncarbonyl oxygen. It should be emphasized that a new peak especially for C–N bound should have appeared, however no such peak in C 1s spectra was observed. This is most probably due to its presence at the same binding energy region as that for C-4. Nonetheless, the presence of such amide bound is clearly shown in Figure 9, representing the N1s spectra (described below), with a binding energy of ~400 eV. The intensity of C-2 peak of grafted ONC decreases by 31%, 23%, 19%, and 11% for ONC-WRF, ONC-Trp, ONC-Phe and ONC-EMP, respectively, compared to that of ONC. This decrease is most probably due to the elimination of hydroxyl groups of the ONC (ester formation) during the fist stage of the coupling reaction of ONC with the amino group of the amino acid or peptide (see experimental section). However, the other C1s peaks remain almost unchanged for all the samples.

**Figure 8:**
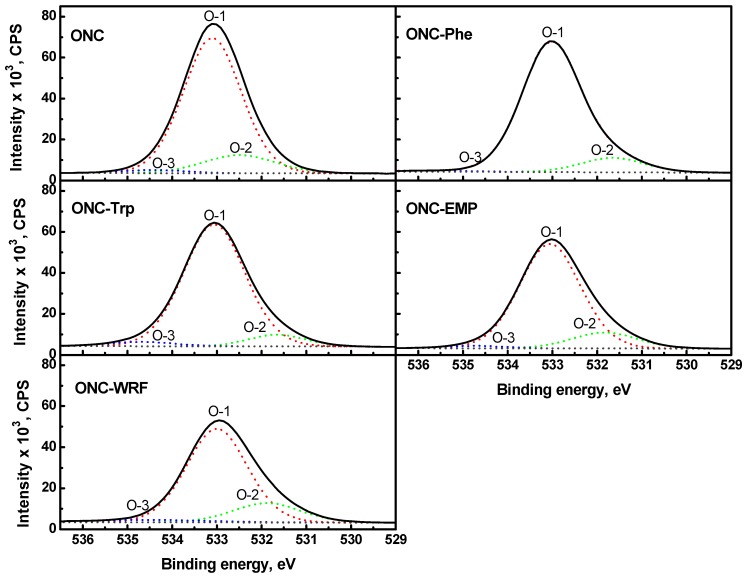
O 1s narrow scan XPS spectra for ONC, ONC-Phe, ONC-EMP, ONC-Trp and ONC-WRF.

Figure 8 presents the high-resolution scans of the XPS spectra of O1s levels with their decomposition into three components. The O-1 peak (~533 eV) for all samples is attributed to an oxygen atom linked to a carbon atom by a single bond. The O-2 peak for ONC sample (~532.5 eV) represents an oxygen atom linked by a double bond to a carbon atom of a carboxylic group. For grafted ONC samples, the O-2 peak (~531.6–531.8 eV) is attributed to an oxygen atom linked by a double bond to a carbon atom of the amide group formed upon coupling reaction. The less intense O-3 peak at higher binding energy (~534.3–535.3 eV) can be attributed to adsorbed water. 

**Figure 9:**
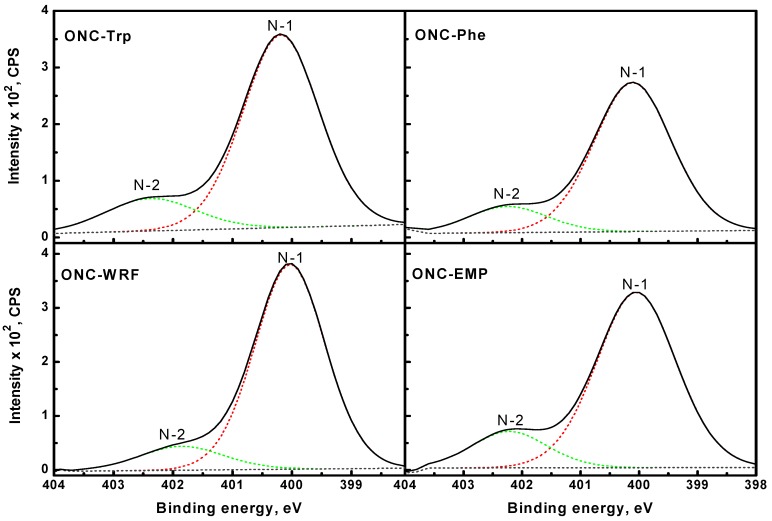
N 1s narrow scan XPS spectra for ONC-Phe, ONC-EMP, ONC-Trp and ONC-WRF.

The deconvolution of N1s levels shown in Figure 9 reveals the presence of two components N-1 and N-2. The N-1 peak (~400 eV) is attributed to a nitrogen atom linked to a carbon atom by a single bond (amide bond) [48], and the N-2 peak (~402 eV) is assigned to protonated amino groups (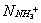) [49]. It should be mentioned that XPS spectra of ONC alone, ONC-amino acids and ONC-Trp-Ps in the absence of coupling agents EDAC-NHS did not show any N 1s signal. 

## 3. Experimental Section

**Materials.** A commercial never-dried bleached kraft pulp was used as native cellulose fibers. The chemicals and solvents used were of the purest quality available purchased from Aldrich Chemical Co. The amino acids and peptides were purchased from American Peptide Company. All the chemicals were used as received.

**Synthesis of oxidized cellulose.** The synthesis procedure of oxidized cellulose is similar to that proposed by Saito *et al.*, [11]. The never-dried kraft pulp (~4 g of cellulose content) was suspended in deionized water (400 mL) containing TEMPO (0.2 g) and sodium bromide (1.2 g). A solution of 13% NaClO was added dropwise to the mixture at room temperature under gentle agitation. The pH was maintained at 10.5 by adding 0.5 M NaOH. When the pH was stable, the reaction was stopped by the addition of methanol (40 mL) and the pH was adjusted to 7 by adding 0.5 M HCl. The TEMPO-oxidized product was filtered, washed thoroughly with deionized water and stored at 4 °C. In the present study, the carboxylate content of the TEMPO-oxidized cellulose was determined using an electric conductivity titration method [12] and it was equal to ~1.2 mmol/g. The sodium carboxylate groups in the TEMPO-oxidized celluloses were converted to free carboxyl ones by ion-exchange treatment; 0.1% cellulose/water slurries were adjusted to pH 2–3 with 0.1 M HCl and left at rest for 1 h followed by washing thoroughly with deionized water. The obtained carboxylated celluloses were used in preparing the oxidized nanocelluloses. 

**Preparation of oxidized nanocellulose (ONC) suspension.** Suspension of transparent ONC was obtained using mechanical disintegration of the oxidized cellulose followed by centrifugation. A mixture of oxidized cellulose (~2g) and 100 mL of deionized water was homogenized with a domestic blender for 20 min at high speed. The blending treatment, although efficient to disrupt the association of microfibrils of the oxidized cellulose, depends highly on the organization of microfibrils in the parent tissue. Further, the TEMPO-mediated oxidation helps the disintegration process by loosening the adhesion between microfibrils and electrostatic repulsions between microfibrils, where significant amounts of carboxylate groups were introduced [11,50]. The obtained slurry was centrifuged at 12,000 g in order to separate oxidized nanocelluloses (supernatant) from large particles.

**Coupling of ONC with amino acid and Trp-based peptide (Trp-Pep).** One of the universal methods for attaching amino acids and peptides or proteins to other materials is diimide-activated amidation. Coupling agents such as EDAC or *N,N’*-dicyclohexylcarbodiimide (DCC) are usually used [25]. However, this process leads to undesirable side reactions of intermolecular connection of peptides since most of them are rich in both amine groups and carboxylic acid groups on their surface. In this study, the intermolecular connection of Trp-based peptides was avoided using a two-step process. 

The coupling of ONC with amino acids and Trp-Pep is a modified version [51] of the two-step attachment procedure proposed by Jiang *et al.*, [25]. The two-step method was carried out at room temperature. In the first step, 5 mL of the ONC suspension (~2.5 mg of dried ONC) were added to 4 mL of deionized water under moderate magnetic stirring. Then, 2.3 mL of a 50 mg·mL^−1^ *N*-hydroxysuccinimide (NHS) aqueous solution were added to the above suspension and mixed. Under fast stirring, 1.2 mL fresh *N*-ethyl-*N’*-(3-dimethylaminopropyl) carbodiimide hydrochloride (EDAC) aqueous solution (10 mg·mL^−1^) was added quickly, and the mixture was continually stirred at room temperature for 30 min while keeping the pH at 7.5–8 by adding 0.5 M NaOH and/or HCl (~1.5–2 mL). The suspension was then dialyzed in deionized water for 8 h to remove excess EDAC, NHS and byproduct urea. The deionized water used for dialysis was changed every hour.

In the second step, 1 mL of a 10 mg·mL^−^^1^ amino acid in deionized water was added to 9 mL of the estered ONC, and for the peptide samples 1 mL of a 1 mg·mL^−1^ peptide in deionized water was added to 14 mL of the estered ONC under moderate magnetic stirring overnight at room temperature. The suspension was then transferred to a dialysis membrane and dialyzed thoroughly in deionized water for 72 h to remove unbound amino acid or peptide. The deionized water used for dialysis was changed every 6h or 8 h. 

**Absorption and emission spectrometry.** Absorption spectra were recorded with a Cary 5000 spectrophotometer. The emission spectra were recorded with a FluoroLog-3 spectrofluorometer.

**Transmission electron microscopy (TEM).** Drops of the suspensions were deposited onto glow-discharged carbon-coated electron microscopy grids. The excess liquid was absorbed by a piece of filter paper, and a drop of 2% uranyl acetate negative stain was added before drying. The liquid in excess was wiped off, and the remaining film of stain was allowed to dry. The specimens were observed using a Philips EM 208S microscope operating at 80 kV. 

**Fourier transform infrared spectrometry (FTIR).** 1 mL of the sample was dried in vacuum dissicator over night.The dried sample was then mixed with KBr and pellets of the mixture were made. FTIR spectra were recorded using a Perkin-Elmer System 2000 in transmission mode. A total of 64 scans were taken per sample with a resolution of 4 cm^−1^ (4000–400 cm^−1^).

**X-Ray photoemission spectroscopy (XPS).** XPS was performed on a Kratos Axis Ultra spectrometer (Kratos Analytical Ltd., UK), using a monochromatic Al K*α* X-ray source (E = 1486.6 eV) with a power of 225 W, at a take-off angle of 90^o^ relative to the sample surface. 400 μL of the sample was dropped on an aluminum substrate and dried in vacuum dissicator overnight to obtain a thin film.The dried sample was then transferred to the XPS sample holder. The measurements were made under a high vacuum of 10^−9 ^torr, at room temperature. The surface of the sample was ~20 mm^2^, and the investigated area was typically 1 × 2 mm^2^. Survey spectra for each sample over a binding energy range of 0–1300 eV were an average of three scans (at three different points) acquired at pass energy of 160 eV and resolution of 1 eV/step (lens in hybrid mode, which assures maximum sensitivity). High-resolution spectra of C 1s, N 1s and O 1s were an average of five scans acquired at a pass energy of 40 eV and resolution of 0.1 eV/step, for quantitative measurements of binding energy and atomic concentration. Because of the potential degradation of the surface during X-ray exposure, the spectra were collected in the same order (survey, C 1s, O 1s, N 1s) such that the amount of exposure to X-rays was equivalent for all analyzed samples. The CasaXPS software was used for background subtraction (Shirley-type), peak integration, fitting and quantitative chemical analysis. The C 1s (C–C) peak at 285 eV was used to calibrate the binding energy scale. Binding energies values are given at ±0.2 eV. Gaussian peak profiles were used for spectral deconvolution of C 1s, O 1s and N 1s spectra. 

## 4. Conclusions

We have synthesized and grafted oxidized nanocellulose (ONC) with amino acids and Trp-based peptides (Trp-Ps) using a simple two-step method. The coupling reaction between ONC and the amino acid or peptide is pH dependent and the grafting was favorable in the pH range 7–10. The TEM images of all samples reveal that the morphology of ONC fibers did not change after their grafting. We have also confirmed the grafting of ONC with amino acids and peptides spectroscopically. In fact, ONC alone does not show any characteristic absorption band and does not fluoresce but after its coupling with amino acids or Trp-Ps, ONC clearly shows the characteristic absorption and fluorescence features of the grafted molecule. The formation of amide bond as a result of coupling between ONC and amino acid or peptide is evidenced by the presence of a small shoulder at 1545 cm^−1^ in FTIR experiment. This absorption is characteristic of the C–N bond of the amide. Finally, the XPS studies further confirmed that N-1 peak (~400 eV) of grafted ONC is due to the nitrogen atom of the amide bond formed between ONC and amino acid or peptide. This coupling approach can also be applied to attach biomolecules (proteins) and other useful molecules containing amine groups, such as polymers and inorganique nanoparticles, onto ONC. 
